# Decreased Influenza Incidence under COVID-19 Control Measures, Singapore

**DOI:** 10.3201/eid2608.201229

**Published:** 2020-08

**Authors:** Roy Jiunn Jye Soo, Calvin J. Chiew, Stefan Ma, Rachael Pung, Vernon Lee

**Affiliations:** Ministry of Health, Singapore (R.J.J. Soo, C.J. Chiew, S. Ma, R. Pung, V. Lee);; Saw Swee Hock School of Public Health, Singapore (V. Lee)

**Keywords:** coronavirus disease, SARS-CoV-2, severe acute respiratory syndrome coronavirus 2, viruses, respiratory infections, zoonoses, coronavirus disease, COVID-19, preventive medicine, infectious disease, Singapore

## Abstract

We compared indicators of influenza activity in 2020 before and after public health measures were taken to reduce coronavirus disease (COVID-19) with the corresponding indicators from 3 preceding years. Influenza activity declined substantially, suggesting that the measures taken for COVID-19 were effective in reducing spread of other viral respiratory diseases.

Public health measures, including public education and physical distancing, were implemented in Singapore to reduce transmission of coronavirus disease (COVID-19). However, instead of a lockdown, Singapore kept schools and workplaces open and did not advise the routine use of masks for persons who are well during the initial phase of the outbreak in January–February 2020. We examined the effect of these COVID-19 measures on influenza incidence as a proxy to determine the overall potential reduction in respiratory virus transmission.

We obtained routine sentinel surveillance data on influenza-like illnesses (ILI) from a national network of primary care clinics and the National Public Health Laboratory. ILI was defined as fever (>38°C) and cough. Data included number of visits to government primary care clinics for ILI per day, ILI samples tested per week, and percentage influenza positivity. We estimated number of influenza cases per day by multiplying ILI visits per day by the proportion of ILI patients who tested positive for influenza, which better reflects influenza infection rates than either indicator alone (*1*).

We compared influenza activity between epidemiologic weeks 1–4 and weeks 5–9 of 2020. Most community-based COVID-19 measures were instituted after the first few cases were reported in epidemiologic week 4, and public awareness was increased (*2*). These measures included cancellation of large-scale events and precautions at schools (e.g., fewer assemblies, no interclass mixing, and staggered meal times) and workplaces (e.g., segregated teams and teleworking wherever possible). Intensive public education on personal hygiene and social responsibility included encouraging regular handwashing and seeking medical attention early when ill. To enable self-isolation and prevent spread, physicians were instructed to give certificates for 5 days of medical leave for patients who had respiratory symptoms but did not meet the COVID-19 suspect case definition.

We compared indicators of influenza transmissibility in 2020 against the average from corresponding periods in the 3 preceding epidemiologic years (2016–2019). We performed weekly paired difference *t*-test using R version 3.5.1 (https://www.r-project.org). Influenza activity peaked in epidemiologic week 1 of 2020 but declined to below the average of the preceding years by epidemiologic week 5 (Figure). Percentage influenza positivity decreased by 64% (p = 0.001) and estimated daily number of influenza cases decreased by 76% (p = 0.002) in epidemiologic weeks 5–9 of 2020 compared with the preceding years. In contrast, we saw no significant changes in any of the indicators, except percentage of influenza positivity (31%; p = 0.008), in epidemiologic weeks 1–4 of 2020 compared with preceding years.

**Figure:**
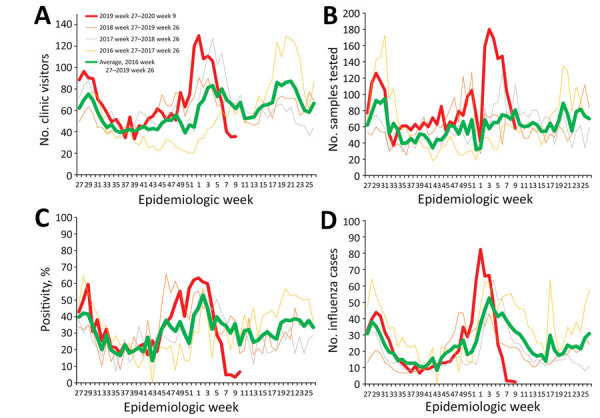
Indicators of influenza activity during the 2019–20 season (red line) compared with average of the preceding 3 years (green line), Singapore. A) Average number of visits per day to government primary care clinics for influenza-like illnesses, 2016–2020. B) Samples from patients with influenza-like illness tested per week, 2016–2020. C) Influenza positivity, 2016–2020. D) Estimated daily numbers of influenza cases, 2016–2020.

Public health efforts to control COVID-19 probably reduced influenza transmission in February 2020 because both viruses have similar modes of transmission through respiratory and contact routes. Even if COVID-19 has a potentially higher basic reproduction number (R_0_) than seasonal influenza (*3*), substantial reduction in transmission could reduce the impact of COVID-19 on healthcare capacity, thereby preventing excess deaths. Our modeling of the effective reproduction number for COVID-19 in Singapore in February 2020 (R. Pung, unpub. data) at 0.5–1 is 55%–77% lower than the mean estimated R_0_ of 2.2 (*4*). This finding is consistent with the observed 76% reduction in influenza transmission.

Our study has several limitations. First, a decrease in influenza transmission is expected in February–March, given the yearly bimodal pattern of influenza incidence in Singapore (*5*). However, the decrease in 2020 is marked compared to previous years. Second, there could be fewer ILI visits to government clinics because of altered health-seeking behavior, or cases may be referred to hospitals and therefore not captured as ILI cases in clinics. However, these missed ILI cases would not affect the proportion positive for influenza. Third, we can infer similar effects on COVID-19 only if the transmission dynamics are similar to influenza.

In conclusion, we found a marked decline in ILI in Singapore after the implementation of public health measures for COVID-19. Our findings suggest that such measures are effective in reducing spread of viral respiratory diseases and could mitigate the impact of the COVID-19 pandemic.
